# Chemopreventive Effects of Concomitant or Individual Use of Statins, Aspirin, Metformin, and Angiotensin Drugs: A Study Using Claims Data of 23 Million Individuals

**DOI:** 10.3390/cancers14051211

**Published:** 2022-02-25

**Authors:** Ching-Huan Wang, Chih-Wei Huang, Phung Anh Nguyen, Ming-Chin Lin, Chih-Yang Yeh, Md. Mohaimenul Islam, Annisa Ristya Rahmanti, Hsuan-Chia Yang

**Affiliations:** 1 Graduate Institute of Biomedical Informatics, College of Medical Science and Technology, Taipei Medical University, Taipei 106339, Taiwan; m301099022@tmu.edu.tw (C.-H.W.); arbiter@tmu.edu.tw (M.-C.L.); d610104001@tmu.edu.tw (C.-Y.Y.); annisaristya@ugm.ac.id (A.R.R.); 2 International Center for Health Information Technology (ICHIT), College of Medical Science and Technology, Taipei Medical University, Taipei 106339, Taiwan; gracehuang@tmu.edu.tw (C.-W.H.); d610106004@tmu.edu.tw (M.M.I.); 3 Clinical Big Data Center, Office of Data Science, Taipei Medical University, Taipei 106339, Taiwan; alexnthhp@tmu.edu.tw; 4 Clinical Big Data Research Center, Taipei Medical University Hospital, Taipei 110301, Taiwan; 5 Department of Healthcare Information and Management, School of Health Technology, Ming Chuan University, Taoyuan 333321, Taiwan; 6 Department of Neurosurgery, Shuang Ho Hospital, Taipei Medical University, New Taipei City 235041, Taiwan; 7 Taipei Neuroscience Institute, Taipei Medical University, Taipei 110301, Taiwan; 8 AESOP Technology, Taipei 105076, Taiwan; 9 Research Center of Big Data and Meta-Analysis, Wan Fang Hospital, Taipei Medical University, Taipei 116079, Taiwan

**Keywords:** chemoprevention, cancer prevention, repurposing, synergistic effect, statin, aspirin, metformin, ACEI, ARB

## Abstract

**Simple Summary:**

Medications for chronic diseases, such as tatins, aspirin, metformin, and angiotensin-converting-enzyme inhibitors/angiotensin II receptor blockers, are studied for decades, and a vast body of previous research has suggested potential repurposing of these medications for cancer prevention. However, a limited number of studies have analyzed the effect of various combinations of these four medications/classes on cancer risks. This study, using large databases, aimed to comprehensively analyze the composite chemopreventive effects of all possible combinations of these four medications/classes. We found no synergistic effect of multiple use of these agents on cancer prevention in this study. Our results may provide information or inspiration for researchers or clinicians to conduct future research on combinations with cancer-preventive potential to further determine the optimal strategy for chemoprevention against cancer.

**Abstract:**

Despite previous studies on statins, aspirin, metformin, and angiotensin-converting-enzyme inhibitors (ACEIs)/angiotensin II receptor blockers (ARBs), little has been studied about all their possible combinations for chemoprevention against cancers. This study aimed to comprehensively analyze the composite chemopreventive effects of all the combinations. In this case-control study, health records were retrieved from claims databases of Taiwan’s Health and Welfare Data Science Center. Eligible cases were matched at a 1:4 ratio with controls for age and sex. Both cases and controls were categorized into 16 exposure groups based on medication use. A total of 601,733 cancer cases were identified. Cancer risks (denoted by adjusted odds ratio; 99% confidence interval) were found to be significantly decreased: overall risk of all cancers in statin-alone (0.864; 0.843, 0.886), aspirin-alone (0.949; 0.939, 0.958), and ACEIs/ARBs (0.982; 0.978, 0.985) users; prostate (0.924; 0.889, 0.962) and female breast (0.967; 0.936, 1.000) cancers in metformin-alone users; gastrointestinal, lung, and liver cancers in aspirin and/or ACEIs/ARBs users; and liver cancer (0.433; 0.398, 0.471) in statin users. In conclusion, the results found no synergistic effect of multiple use of these agents on cancer prevention. Use of two (statins and aspirin, statins and metformin, statins and ACEIs/ARBs, and aspirin and ACEIS/ARBs) showed chemopreventive effects in some combinations, while the use of four, in general, did not.

## 1. Introduction

In 2020, globally, cancer was newly diagnosed in an estimated 19.3 million cases and led to 10.0 million deaths [1]. Of all sites, female breast cancer accounted for the highest proportion of new cancer cases at 11.7%, followed by lung, colorectal, prostate, and stomach cancers [1]. Meanwhile, lung cancer was the leading cause of deaths (18.0%) among all cancer sites, preceding colorectal, liver, stomach, and female breast cancers [1]. Recent years have seen research on repurposing chronic disease medications for chemoprevention.

Common medications for chronic diseases, such as the following agents, have been marketed and extensively studied for decades: β-hydroxy β-methylglutaryl-coenzyme A reductase inhibitors (HMG-CoA inhibitors, “statins”), aspirin, metformin, angiotensin-converting-enzyme inhibitors (ACEIs), and angiotensin receptor blockers (ARBs). Statins, as a class of lipid-lowering agents used for hypercholesterolemia treatment and cardiovascular disease (CVD) prophylaxis [2], are found to be associated with reduced risks of hepatic [3] and pancreatic [2,4] cancers. The prostate cancer risk among statin users, as opposed to non-users, remains inconclusive, suggested to be reduced [5], increased [6], or not significantly associated with use of statins [7]. Aspirin, a cyclooxygenase and thromboxane inhibitor indicated for pain, inflammation, and CVDs, has shown associations with lowered risks of gastrointestinal cancers, including esophageal [8,9], stomach [10], liver [11], and colorectal [12] cancers. The chemopreventive effect of aspirin against pancreatic cancer could be nonsignificant [13,14] or marginal [15] in general, albeit significant in postmenopausal women [16]. Metformin is an anti-hyperglycemic agent prescribed for type 2 diabetes mellitus [2], known in recent years for its negative association with cancer risks of various sites, such as head and neck [17], lung [18], breast [19], liver [20,21], pancreas [2], colorectum [19], and debatably, prostate, with conflicting results published in previous studies [6,7]. ACEIs and ARBs (ACEIs/ARBs) exert an inhibitory effect on the renin-angiotensin-aldosterone system (RAAS), approved for CVDs or diabetic nephropathy. Despite being equivocal, reduced risks of cancers among ACEI/ARB users have been proposed, such as colorectal [22,23] and overall [24] cancer risks.

Although a vast body of research has been conducted on repurposing of the aforementioned four medications or classes for cancer prevention, previous studies predominantly focused either on a single class of medications or on a certain combination of different pharmacological classes. In other words, little has been studied about all possible combinations of these four medications or classes. The aim of this study, therefore, was to comprehensively analyze the composite chemopreventive effects of all possible combinations of these four medications/classes. This study may provide information or inspiration for researchers or clinicians to determine the optimal strategy for chemoprevention against cancer for patients.

## 2. Methods

### 2.1. Data Sources

To conduct this case-control study, we retrieved health records from databases of Health and Welfare Data Science Center (HWDC) administered by Taiwan’s Ministry of Health and Welfare (MOHW). These databases have stored de-identified claims data of beneficiaries of the National Health Insurance [25], which is a compulsory insurance covering a population of over 23 million, namely 99.8% of residents in Taiwan [26]. This study has been approved by the Joint Institutional Review Board of Taipei Medical University (TMU-JIRB), Taipei, Taiwan (approval numbers: N201602065 and N202003069, 23 March 2016). All the data analyzed in this study were de-identified, thereby rendering informed consent impossible and waived.

### 2.2. Study Design

**Figure 1:**
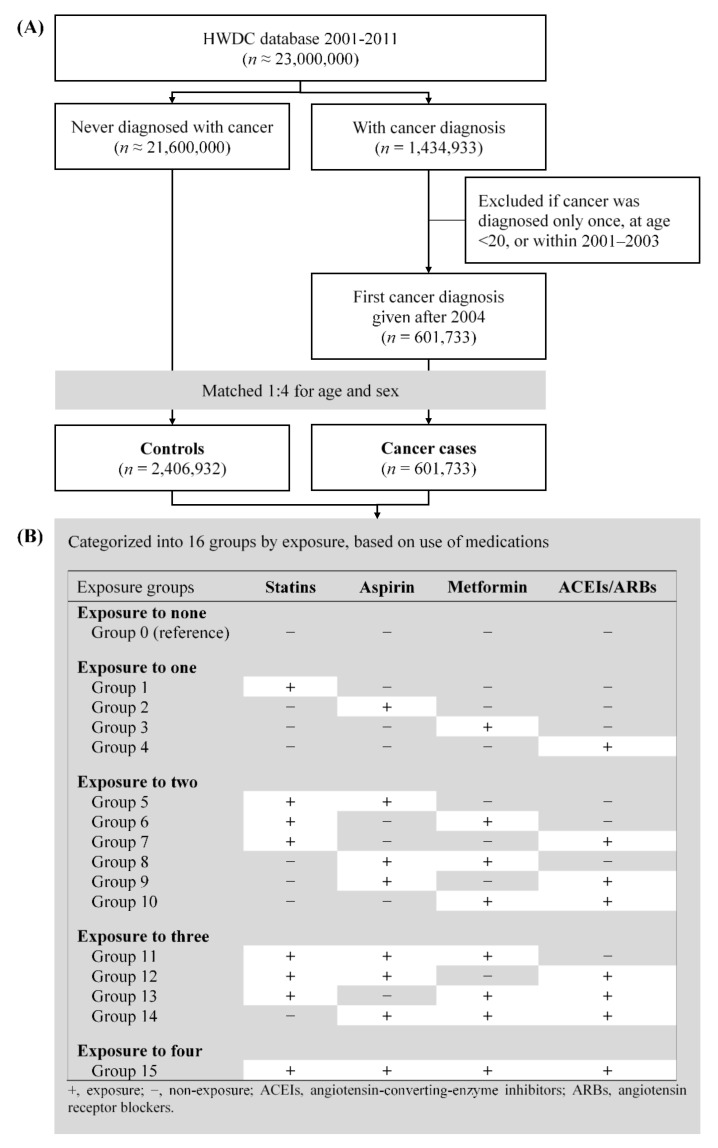
Study design. (**A**) Process of identifying cancer cases and controls. (**B**) Categorization of cases and controls into 16 groups based on use of statins, aspirin, metformin, and ACEIs/ARBs.

We enrolled data of prescriptions and diagnoses given during the study period from 1 January 2001 through 31 December 2011 (Figure 1A). Patients who had had their first cancer diagnosis during the study period were selected from the total population of all beneficiaries in the databases. Patients diagnosed with cancer between 2001 and 2003 were excluded from analysis given this study’s definition of exposure to the common medications—statins, aspirin, metformin, and ACEIs/ARBs—for chronic diseases. Eligible cases were subsequently matched with controls for age and sex at a 1:4 ratio to improve the power of our study [27]. The index date was defined, for cases as the date of the first cancer diagnosis, and for controls as the same index date as their matched cases.

Both cases and controls were categorized into 16 exposure groups (Figure 1B), including the non-exposure group, based on use of statins, aspirin, metformin, or ACEIs/ARBs within 3 years of the index date. Patients exposed to none of the four medications/classes were the reference group (the non-exposure group, Group 0). The other 15 exposure groups consisted of those exposed to one (Groups 1–4), two (Groups 5–10), three (Groups 11–14), and all (Group 15) of the four medications/classes.

### 2.3. Definitions of Cases and Controls

Cases in this study had to meet the inclusion criteria as follows: having the first diagnosis of primary cancer after 1 January 2004, aged 20 or older at diagnosis of cancer, and diagnosed with a certain cancer at least twice in order to confirm the cancer diagnosis. Eligible for controls were those who had had no cancer history within the study period. Diagnoses were encoded based on the International Classification of Diseases, 9th revision, Clinical Modification (ICD-9-CM). Risk of cancers evaluated in this study are as follows (ICD-9-CM-codes listed in parentheses): overall risk of cancers (140.xx–208.xx), female breast cancer (174.xx), prostate cancer (185.xx), lung cancer (172.xx–173.xx), liver cancer (162.xx), and gastrointestinal cancers (150.xx–154.xx).

### 2.4. Definition of Exposure

Considering a long period of time required for cancer development, along with adequate exposure to the four medications/classes needed to exert an effect on cancer risk, we defined exposure to a medication as prescription of the medication for at least 60 days within three years prior to the index date. Medications were encoded by the World Health Organization’s Anatomical Therapeutic Chemical (ATC) classification system, namely statins (C10AA), aspirin (B01AC06), metformin (A10BA02), ACEIs (C09A), and ARBs (C09C). ATC codes of combination products were broken down into codes of single active ingredients.

### 2.5. Potential Confounding Factors

Comorbidities were considered to be potential confounders in this study. Outcomes of this study were adjusted for both age-adjusted Charlson comorbidity index (ACCI) score and its components listed as follows with their ICD-9-CM codes in parentheses: myocardial infarction (410.x and 412), congestive heart failure (428.x), peripheral vascular disease (441.x, 443.9, 785.4, and V43.4), cerebrovascular disease (430–438), dementia (290.x), chronic pulmonary disease (490.x–496.x, 500.x–505.x, and 506.4), rheumatologic disease (710.0, 710.1, 710.4, 714.0–714.2, 714.81, and 725.x), peptic ulcer disease (531–534.9), liver disease (456.0–456.21, 571.2, 571.4–571.6, and 572.2–572.8), diabetes mellitus (250.x), hemiplegia or paraplegia (342.x and 344.1), and renal disease (582.x, 583.0–583.7, 585.x, 586.x, and 588.x) [28].

### 2.6. Statistical Analysis

We analyzed cancer risks of each exposure group using conditional logistic regression as the formula below [29,30]:

$$\mathrm{Conditional}\;\mathrm{likelihood}=\overset{n}{\underset{i=1}{\prod }}\frac{\exp\left(\beta_{1}X_{i01}+\cdots +\beta_{k}X_{i0k}\right)}{\sum_{j=0}^{m}\exp\left(\beta_{1}X_{ij1}+\cdots +\beta_{k}X_{ijk}\right)}$$

where the *i*th case of a total of *n* cases is matched with m controls (*m* = 4), *β* is the regression coefficient of each predictor *X*, and *k* is the number of predictors (*k* = 16) consisting of age, sex, exposure group, and comorbidities. Each of the 15 exposure groups was individually compared with the reference group, and cancer risks were estimated as odds ratios adjusted for age, sex, and comorbidities (aORs), namely exp(*β*). A *p*-value ≤ 0.01 for cancer risk estimation was regarded as statistically significant [31]. A 99% confidence interval was estimated for each aOR. All statistical tests in this study were two-tailed. The SAS software (version 9.4; SAS Institute Inc., Cary, NC, USA) was used for statistical analysis.

## 3. Results

### 3.1. Demographic Characteristics

A total of 601,733 cancer cases within the study period were identified and matched with 2,406,932 controls (Table 1). Both cancer cases and controls had an average age of 60.47 years, with ACCI scores of 3.58 and 3.31, respectively. A slight majority (53.96%) of both these populations were males. Of all cancer cases, 33.61% (*n* = 202,224) had used any of the four medications/classes for at least 60 days within the three years prior to a cancer diagnosis, and close to 33.09% in controls (*n* = 796,532).

### 3.2. Prescription Patterns of the Four Medications/Classes among Cases and Controls

Table 2 presents the percentage of each exposure group in cases and controls of all cancers, prostate cancer, female breast cancer, liver cancer, GI cancer, and lung cancers. Patients using none of the four medications/classes of interest (Group 0, the reference group) accounted for a noticeable 78.79% and 52.10% of female breast cancer and prostate cancer cases respectively, as compared to 66.39% for the overall cancer population. Among the 15 exposure groups, ACEIs/ARBs (Group 4) were the most commonly prescribed medications, irrespective of cancer types, making up 7.73% of all cancer cases, 4.84% of females with breast cancer, and 10.83% of males with prostate cancer. In stark contrast, a mere 0.43% of cases with any type of cancer had used all the three medications—statins, aspirin, and metformin (Group 11)—prior to a cancer diagnosis. 

Among those with a history of using two of the four medications/classes, use of aspirin and ACEIs/ARBs (Group 9) was the most common combination, accounting for 4.16% of the overall cancer cases. As for use of three medications/classes, the combination of statins, aspirin, and ACEIs/ARBs (Group 12) was the most frequent one in general, at 1.88% among all cancer patients, except for liver cancer cases where the combined use of aspirin, metformin, and ACEIs/ARBs (Group 14) was more common, at 2.09% compared with 1.14% for Group 12.

### 3.3. Chemopreventive Effects of the Four Medications against Cancers

**Figure 2:**
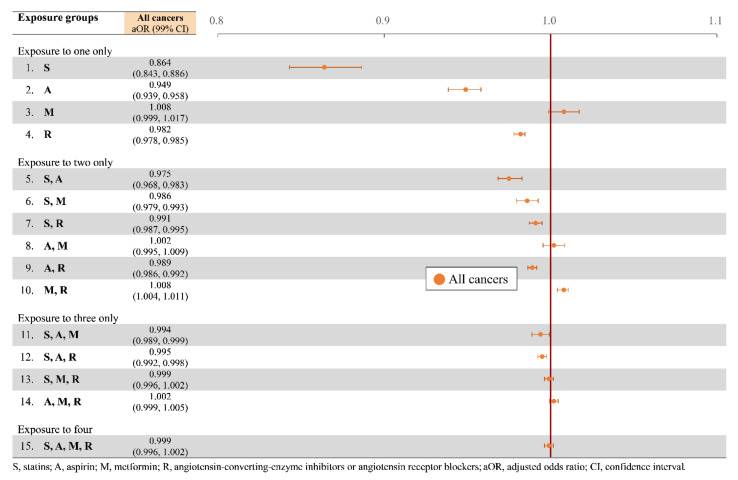
Overall risk of all cancers in adjusted odds ratio.

Summarized in Table 3 are statistically significant increases and decreases in risks of all cancers, prostate cancer, female breast cancer, liver cancer, GI cancers, and lung cancers among the exposure groups. Use of statins, aspirin, or ACEIs/ARBs alone was associated with a significantly decreased aOR of the overall cancer risk—hereinafter expressed through “aOR (the lower bound, the upper bound of the 99% confidence interval (99% CI))”—at 0.864 (0.843, 0.886) for statins, 0.949 (0.939, 0.958) for aspirin, and 0.982 (0.978, 0.985) for ACEIs/ARBs (Figure 2). 

Meanwhile, the statin-alone group demonstrated a marginal increase in the overall cancer risk, with an aOR of 1.008 (0.999, 1.107), a result consistent with an aOR of 0.995 (0.992, 0.998) for combined use of statins, aspirin, and ACEIs/ARBs. Use of more than one of these medications, despite failing to show a lower risk than use of a single medication did, still indicated a significantly decreased overall risk of all cancers in certain combinations. The aORs of all cancers were 0.975 for previous exposure to statins and aspirin, 0.986 for statins and metformin, 0.991 for statins and ACEIs/ARBs, 0.989 for aspirin and metformin, 0.994 for statins and aspirin and metformin, and 0.995 for statins and aspirin and ACEIs/ARBs.

**Figure 3:**
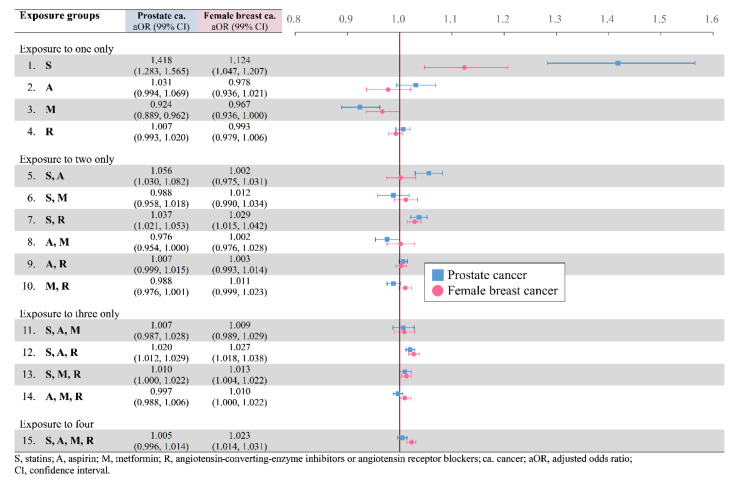
Prostate and female breast cancer risks in adjusted odds ratio.

For common sex-specific cancers, use of metformin either alone or combined with aspirin was associated with a lowered risk of prostate cancer, with aORs of 0.924 and of 0.976, respectively; in addition, the metformin-alone group was the only one group that demonstrated a decreased risk of female breast cancer, with an aOR of 0.967 (Figure 3).

**Figure 4:**
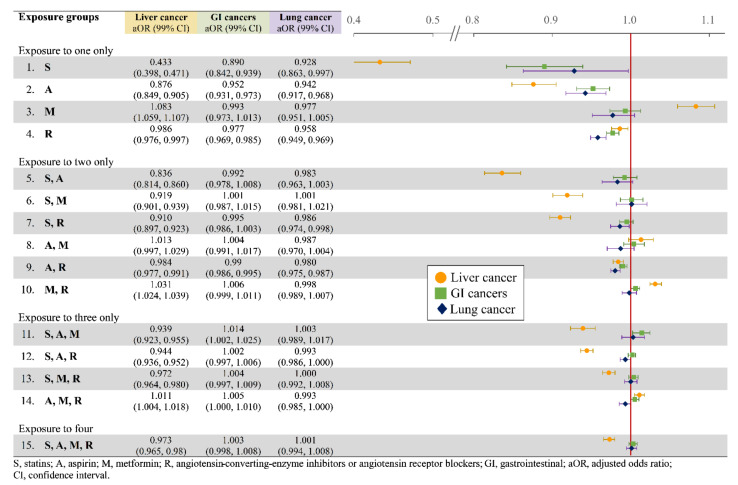
Liver, lung, and gastrointestinal cancer risks in adjusted ratio.

A significantly decreased risk of liver cancer was found in users of statins, aspirin, or ACEIs/ARBs alone, especially a remarkable aOR of 0.433 for statin users, much lower than 0.876 for aspirin and 0.986 for ACEI/ARB users (Figure 4). Meanwhile, metformin-alone users were more likely to develop liver cancer, with an aOR of 1.083. When combined, the medications/classes demonstrated a composite effect against liver cancer, plausibly counterbalancing each other.

Similar to the overall cancer risk, no synergistic pattern was observed in risks of lung and gastrointestinal cancers. Risks of lung and gastrointestinal cancers were significantly decreased in users of statins, aspirin, and ACEIs/ARBs alone, with aORs of 0.928, 0.942, and 0.958 for lung cancer and 0.890, 0.952, and 0.977 for gastrointestinal cancers, respectively (Figure 4). Combination of two or three of these medications, however, was not necessarily associated with lowered cancer risk.

## 4. Discussion

The rate of exposure to any of the four medications/classes was lowest in female cases with breast cancer and highest in male cases with prostate cancer. ACEIs/ARBs were the class most commonly prescribed among the four medications/classes. In general, the results showed no decrease in cancer risks with the increased number of the medications/classes used, indicating possibly no synergistic effect of multiple use of statins, aspirin, metformin, and/or ACEIs/ARBs for cancer prevention. Metformin was found to have a preventive effect against prostate and female breast cancers, whereas aspirin and/or ACEIs/ARBs demonstrated reduced risks of gastrointestinal, lung, and liver cancers. Additionally, statins showed a noticeable decrease in the risk of liver cancer.

### 4.1. Prescription Patterns

The prescription patterns can be explained in part by the prevalence of the cancers and of chronic diseases for which the four medications/classes are indicated. The difference in exposure to any of the four medications/classes between female breast cancer and prostate cancer could be attributed to patients’ age at cancer diagnosis. In clear contrast to a median age of 56 for female breast cancer occurrence, prostate cancer occurs at a median age of 72 [32]. Given the prevalence rates of chronic diseases increasing with age [33], it is reasonable that a relatively low number of female breast cancer cases and a relatively high proportion of prostate cancer cases had been exposed to any of the four medications/classes.

In Taiwan, the prevalence rate of hypertension in elderly residents (aged 65 or more) is 63.52%, higher than 37.90% for hyperlipidemia and 27.84% for hyperglycemia [33]. In our study, despite a relatively low average age of 60.47, the distinctive high rate of using the antihypertensive agents—ACEIs/ARBs—among the four medications/classes is consistent with the high prevalence of hypertension in the elderly.

### 4.2. Postulated Mechanisms of the Four Medications/Classes against Cancers

Mechanisms of metformin’s antineoplastic effects against prostate have been proposed, including suppression of signaling in insulin-like growth factor-1 (IGF-1), androgen receptor (AR), phosphatidylinositol 3-kinase (PI3K)/protein kinase B (AKT)/mammalian target of rapamycin (mTOR), and hedgehog (Hh) pathways [34]. In addition, metformin has been postulated to inhibit the development of female breast cancer by reducing circulating hormone levels and glycogenesis [35,36].

Statins, considered to have effects against liver cancer, can induce apoptosis and inhibit angiogenesis, proliferation, and invasion of tumor cells by interfering with the downstream products of the mevalonate pathway [37]. Aspirin can reduce vessel formation and triggers apoptosis by reversing the balance of pro- and anti-angiogenetic effects in the tumor microenvironment [38] and modulating both the nuclear factor kappa B (NF-κB) and the signal transducer and activator of transcription 3 (STAT3) pathways [2]. Additionally, activation of type 1 angiotensin II receptors (AT1R) in the RAAS may benefit the establishment of a tumorigenic microenvironment, and ACEIs/ARBs, as RAAS inhibitors, are likely to inhibit expressions of vascular endothelial growth factor (VEGF), matrix metalloproteinase (MMP)-7, tumor-associated macrophages, and *c-myc* [39].

### 4.3. Explanations for Our Findings in This Study

In this study, we found lowered overall risks of cancers in users of statins alone, aspirin alone, and ACEIs/ARBs alone. Japanese randomized controlled trials with 10-year follow-ups in type 2 diabetic patients also revealed significant decreases in incidence of cancers in statin [40] and aspirin users [41]. Besides this, in general, the overall risk of cancers approached an aOR of 1 with the increased number of the four medications/classes combined. This could be explained by the fact that those prescribed with multiple drugs were likely to have more chronic diseases and a generally deteriorating condition as opposed to those treated with a relatively small number of medications. Despite the adjustments for comorbidities, chemopreventive effects of the four medications/classes could be weakened by patients’ relatively poor health conditions.

Supported by a Korean study based on claims data of a large population [7], our study also found a significantly reduced risk of prostate cancer. A Swedish cohort study, nonetheless, suggested no preventive effect of metformin against prostate cancer [6]. Such a discrepancy in metformin’s effect might be due to the differences in study design and racial distribution as opposed to our study. Partly in line with our results, the Swedish study indicated an elevated high-grade prostate cancer risk in statin users [6]. Statins have shown both promoting and inhibitory effects on prostate carcinogenesis [5], depending on cumulative duration and dose [42].

As for metformin’s potential preventive effect against breast cancer risk observed in this study, there has been a considerable body of in-vivo or in-vitro evidence that accords with our findings [35,36]. In previous studies, a decrease in breast cancer risk has been observed in long-term metformin users [43], with a dose-response relationship [19]. A study, however, indicated that metformin may enhance ER-negative and tri-negative breast cancer risks in females with type 2 diabetes, despite a possible association between long-term use of metformin and a reduced risk of estrogen receptor (ER)-positive breast cancer in this population [44].

Consistent with this study, the negative association between statin use and the liver cancer risk has been identified in previous observational studies [45]. Meanwhile, we observed an elevated risk of liver cancer in metformin users, a finding that conflicts with results of previous studies [46,47]. This inconsistency could be explained in part by the difference in metformin users’ medication history: absence of use of any of statins, aspirin, and ACEIs/ARBs in this study, in contrast to inclusion of metformin users irrespective of use of any of the above-mentioned agents in previous research.

Gastrointestinal cancers were defined in this study as ones developed at esophagus, stomach, intestines, colons, or the rectum. Our findings are supported by original studies and meta-analyses that have indicated reduced incidences of esophageal [48], gastric [10,48,49], and colorectal [48,49,50] cancers in aspirin users and of colorectal cancer in ACEI/ARB users [22,23,51]. 

We identified a lowered lung risk in users of aspirin and/or ACEIs/ARBs. Some studies, nevertheless, showed no significant association between aspirin use and lung cancer risk [18,49]. Our explanation is that aspirin’s chemopreventive effect may possibly be counterbalanced by the increased lung cancer risk in patients using aspirin at a high frequency [49,52], especially in those aged <76 or with a body weight <80 kg [52]. Additionally, ACEIs/ARBs have shown their association with a decreased risk of lung cancer in previous studies [53,54]. Research articles have further indicated a lower incidence of lung cancer in ARB users than in their ACEI counterparts [55,56]. It has, however, been revealed that high cumulative doses of ACEIs/ARBs are associated with an elevated risk of lung cancer [57].

To date, combined use of two or more of statins, aspirin, metformin, and ACEIs/ARBs has not been thoroughly researched. Literature has suggested associations that support our findings in this study, including a reduced liver cancer risk in users of statins plus metformin [20] and in those of statins and aspirin [58]. There are, however, still earlier findings conflicting with ours, such as decreases in liver cancer risk among users of aspirin plus metformin [20] and in lung cancer risk among patients using statins, aspirin, plus metformin [18] identified in previous studies but not in this study. In addition, this study presented associations of cancer risks with various drug combinations that have not been researched previously. Given the limited body of current evidence as well as inconsistent findings, chemopreventive effects of combinations of any of these medications/classes are to be confirmed by future research.

With a large sample size of three million subjects including more than 600,000 cancer cases, this study, to our knowledge, is the first study that analyzes chemopreventive effects of all the 15 possible combinations of statins, aspirin, metformin, and ACEIs/ARBs. This study attempted to comprehensively analyze the effects of all the 15 possible combinations of statins, aspirin, metformin, and ACEIs/ARBs. Rather than determine the causality between risk of a cancer and any of the combinations, this study aimed to preliminarily present associations of these combinations with cancer risks. Our study is expected to provide pharmacoepidemiologic information for researchers interested in repurposing these medications. Based on the findings of this study, promisingly, researchers would be capable of identifying combinations with cancer-preventive potential and conducting future studies on combinations of interest to confirm the causation and the mechanisms, and further determine optimal strategies for cancer prevention.

## 5. Limitations

We, however, have to acknowledge limitations of this study as follows. First of all, our data are supposed to be interpreted with caution. This observational study demonstrated an association—instead of causality—of a combination with a cancer risk. Despite a vast body of research on possible anti-neoplastic effects of any of the four medications/classes alone, hardly can previous studies explain the composite effects of combined use of these medications/classes on cancer risks. Future interventional studies and meta-analyses would be required to confirm the mechanisms and causality of these associations. Second, this study retrieved data from the HWDC databases, which had collected and stored claims data of residents in Taiwan. The study population was predominately Asian, aged approximately 60 on average. Therefore, our results might not be generalizable to certain scenarios or populations given the differences in demographic characteristics. Third, without data of patients’ adherence, exposure to the medications in this study was based on patients’ prescription data, which may not necessarily reflect the real use of the medications. Fourth, we could hardly obtain data from the databases the information, such as lifestyle and laboratory data, that were considered factors for cancer risks; nevertheless, such a gap was narrowed by adjustment for Charlson comorbidities in this study.

## 6. Conclusions

Our results suggested no synergistic effect of multiple use of statins, aspirin, metformin, and/or ACEIs/ARBs for cancer prevention. We found significantly reduced risks of prostate and female breast cancers in metformin-alone users, of gastrointestinal, lung, and liver cancers in aspirin and/or ACEIs/ARBs users, and of liver cancer in statin users. In some combinations, use of two of these medications/classes also showed significant chemopreventive effects. In contrast, no reduction in risks of cancers, except for liver cancer, was identified in users of all the four medications/classes. Future research is required to confirm the causality and mechanisms of the associations identified in this study.

## Figures and Tables

**Table 1:** Demographic characteristics of all cancer cases and controls.

Characteristics	All Cancer Cases (*N* = 601,733)		Controls (*N* = 2,406,932)	
Age (years), mean ± *SD*	60.47	±15.37	60.47	±15.37
Male sex, *n* (%)	324,710	(53.96)	1,298,840	(53.96)
Age-adjusted CCI score, mean ± *SD*	3.58	±2.68	3.31	±2.57
Exposure groups, *n* (%)				
Exposure to none				
0. No exposure (reference group)	399,509	(66.39)	1,610,400	(66.91)
Exposure to one only				
1. Statin	13,966	(2.32)	59,410	(2.47)
2. Aspirin	21,691	(3.60)	92,676	(3.85)
3. Metformin	16,380	(2.72)	58,131	(2.42)
4. ACEIs/ARBs	46,506	(7.73)	190,039	(7.90)
Exposure to two only				
5. Statin and aspirin	6284	(1.04)	26,727	(1.11)
6. Statin and metformin	5602	(0.93)	22,011	(0.91)
7. Statin and ACEIs/ARBs	10,996	(1.83)	43,060	(1.79)
8. Aspirin and metformin	4085	(0.68)	14,585	(0.61)
9. Aspirin and ACEIs/ARBs	25,015	(4.16)	103,603	(4.30)
10. Metformin and ACEIs/ARBs	12,739	(2.12)	42,309	(1.76)
Exposure to three only				
11. Statin, aspirin, and metformin	2580	(0.43)	9934	(0.41)
12. Statin, aspirin, and ACEIs/ARBs	11,311	(1.88)	44,697	(1.86)
13. Statin, metformin, and ACEIs/ARBs	7882	(1.31)	28,571	(1.19)
14. Aspirin, metformin, and ACEIs/ARBs	8833	(1.47)	30,584	(1.27)
Exposure to four				
15. Statin, aspirin, metformin, and ACEIs/ARBs	8354	(1.39)	30,195	(1.25)

ACEIs, angiotensin-converting enzyme inhibitors; ARBs, angiotensin-receptor blockers; CCI, Charlson comorbidities index.

**Table 2:** Distributions of the 16 exposure groups in cancer cases and controls.

Exposure Groups		All Cancers		Prostate Cancer		Female Breast Cancer		Liver Cancer		GI Cancers		Lung Cancer	
		+	−	+	−	+	−	+	−	+	−	+	−
Total, *n*		601,733	2,406,932	32,419	129,676	65,491	261,964	81,207	324,828	121,934	487,736	68,409	273,636
Exposure to none													
0.	Reference, %	66.39	66.91	52.10	54.21	78.79	78.76	63.97	65.22	62.73	62.71	60.60	59.30
Exposure to one only													
1.	S, %	2.32	2.47	3.03	2.12	2.76	2.60	1.51	2.53	2.47	2.58	2.54	2.64
2.	A, %	3.60	3.85	6.30	6.27	1.78	2.00	3.24	3.91	4.08	4.46	5.02	5.13
3.	M, %	2.72	2.42	2.34	2.87	1.55	1.79	5.05	2.68	2.74	2.61	2.45	2.65
4.	R, %	7.73	7.90	10.83	10.73	4.84	5.21	8.90	8.20	8.33	8.85	8.86	9.56
Exposure to two only													
5.	S and A, %	1.04	1.11	1.89	1.52	0.66	0.71	0.56	1.19	1.24	1.25	1.37	1.39
6.	S and M, %	0.93	0.91	0.84	0.86	0.88	0.86	0.86	0.98	1.05	0.97	0.98	1.00
7.	S and R, %	1.83	1.79	2.69	2.07	1.52	1.34	1.18	1.86	2.06	2.02	2.10	2.17
8.	A and M, %	0.68	0.61	0.76	0.93	0.31	0.33	1.00	0.65	0.76	0.70	0.73	0.78
9.	A and R, %	4.16	4.30	7.61	7.54	1.62	1.72	4.17	4.43	4.78	5.09	5.64	5.85
10.	M and R, %	2.12	1.76	2.17	2.37	1.14	1.09	3.59	1.85	2.27	2.01	2.08	2.09
Exposure to three only													
11.	S, A, and M, %	0.43	0.41	0.57	0.52	0.30	0.30	0.33	0.47	0.54	0.44	0.53	0.49
12.	S, A, and R, %	1.88	1.86	3.55	2.95	1.12	0.90	1.14	2.02	2.25	2.15	2.45	2.36
13.	S, M, and R, %	1.31	1.19	1.50	1.26	1.11	1.00	1.23	1.29	1.47	1.29	1.40	1.39
14.	A, M, and R, %	1.47	1.27	1.98	2.08	0.64	0.62	2.09	1.34	1.67	1.48	1.59	1.64
Exposure to four													
15.	>S, A, M, and R, %	1.39	1.25	1.84	1.68	0.97	0.78	1.18	1.39	1.56	1.40	1.67	1.56

S, statins; A, aspirin; M, metformin; R, angiotensin-converting-enzyme inhibitors or angiotensin receptor blockers; +, cancer diagnosis; −, absence of cancer diagnosis; GI, gastrointestinal.

**Table 3:** Summary of significant increases and decreases in cancer risks among the exposure groups.

Exposure Groups		All Cancers	Prostate Cancer	Female Breast Cancer	Liver Cancer	GI Cancers	Lung Cancer
Exposure to none							
0.	Reference, %	(reference)	(reference)	(reference)	(reference)	(reference)	(reference)
Exposure to one only							
1.	S, %	↓	↑	↑	↓	↓	↓
2.	A, %	↓			↓	↓	↓
3.	M, %		↓	↓	↑		
4.	R, %	↓			↓	↓	↓
Exposure to two only							
5.	S and A, %	↓	↑		↓		
6.	S and M, %	↓			↓		
7.	S and R, %	↓	↑	↑	↓		↓
8.	A and M, %		↓				
9.	A and R, %	↓			↓	↓	↓
10.	M and R, %	↑			↑		
Exposure to three only							
11.	S, A, and M, %	↓			↓	↑	
12.	S, A, and R, %	↓	↑	↑	↓		↓
13.	S, M, and R, %			↑	↓		
14.	A, M, and R, %			↑	↑		↓
Exposure to four							
15.	S, A, M, and R, %			↑	↓		

S, statins; A, aspirin; M, metformin; R, angiotensin-converting-enzyme inhibitors or angiotensin receptor blockers; ↑, significant increase; ↓, significant decrease; GI, gastrointestinal.

## Data Availability

Restrictions apply to the availability of these data. Data was obtained from databases of Health and Welfare Data Science Center and are available with the permission of Taiwan’s Ministry of Health and Welfare.
